# Nerve Study in a Nervous Age

**Published:** 1896-11-28

**Authors:** 


### The Hospital

A Journal of

The Medical Sciences and Hospital Administration, with

AN EXTRA NURSING SUPPLEMENT.

Vol. XXI.—No. 531.] [Nov. 28, 1896.

### Nerve Study in a Nervous Age.

That ours is a nervous age every brain-worker knows. Every man is a brain-worker who devotes himself with all his powers to work which demands intelligence. The medical profession and the public, in our nervous age, may congratulate themselves upon the fact that now at length a special medical school for the study of the nervous system and its diseases has been opened in connection with the National Hospital for the Paralysed and Epileptic.

In an opening address, too modestly described by the speaker himself as "commonplace," Dr. W. R. Gowers demonstrated, with a clearness and a force worthy of the best traditions of scientific exposition, the commanding place which the nervous system holds in all the higher animals, but especially in man, and the imperative necessity which a strenuously intellectual epoch imposes upon the healing profession of mastering the structure and functions of that system in all its most complex details, and of studying with the utmost diligence every circumstance which influences it for the worse or the better.

It is a question of great interest to ascertain to what extent the medical profession is likely to avail itself of opportunities for studying diseases of the nervous system. A fact mentioned at the opening of the new medical school enables us to answer this question. It is the case that, during the last nine years, no fewer than 2,500 practitioners and students have availed themselves of the opportunities for study afforded by the wards of the hospital, which now adds a medical school to its functions.

It is not always given to specialists to see the wide bearings of their work, or its broadly human—as distinguished from its more limited professional or scientific—significance. The specialists and the great world too, if they knew the past, or could foresee the future, would look with interested, not to say anxious, eyes upon the opening of this school for the special study of the nervous system. Are the strenuous brain-workers of the present able to bear the strain which they put upon themselves, and to live, with unimpaired mental and bodily vigour, to a normal age? It is the nervous system which must answer this question for us. Will succeeding ages be equal to the strain of an increasingly complex civilisation, or are the suns of British and other civilisations now at their meridians, and must we look for twilight and darkness in Europe and America in the near future? Again it is the human nervous system to which we must look for an answer. Statistics tell us that the number of suicides is steadily on the increase. No man ever yet committed suicide who had a perfectly normal and healthy nervous system. Lunatic asylums are constantly being enlarged and filled with an increasing crowd of unhappy prisoners. It is impaired nervous systems which swell those doleful crowds. "Degeneration" is a word which, more than any other, has appeared in recent books, and arrested attention in the columns of the newspapers. The degeneration which is in every man's mind is chiefly mental and moral degeneration; and the starting point of this, as of other degenerations, is the brain, the physical nervous system. What the soul is to the body, the nervous system is to the life. It is the centre, the vivifier, the ruler, the alpha, and the omega. That general medical science should attack, and attack victoriously, the citadel of the nervous system is one of the most necessary of all the conquests which an invincible human progress has yet to achieve.

The common span of life, we are told, is extended in our epoch. The average human being lives longer than his predecessors, and not only longer, but more prosperously and happily. But at whose expense is this general longevity secured? At the expense of the brain-worker. Does the man of science—and more especially of medical science—live longer, or more pleasantly than before? For answer let us give the names of a Huxley, a Clarke, a Bristow, a Reynolds, a Johnson, a Harley. All of these were scientific men of the first rank. Their average ages were some years under seventy. They all died so recently that the funeral bell of the first hardly ceased to toll before the last was called to join in the mournful train. But what will the unthinking world be and do without its great brain-workers? It is poor consolation to think that the heedless many live on in their prosperous heedlessness, whilst those who discover for them the means of increasing happiness and longer life die before their time. We must find some means of keeping our brain-workers alive. That is one of the medical problems of the hour. Brain-workers are the life and soul of the world. For them life must be made less strenuous, more prosperous, and more joyful, in order that it may become more enduring. The world should value brain-workers as its choicest and most indispensable possessions. It is because we see in the profound and general study of the nervous system the means of conserving the brain, the health, and the noblest capacities of the highest workers of the nation that we welcome, among our other scientific institutions, a "special" medical school for study and instruction in the nervous system.

